# The Traditional Chinese Medicine Kangai Injection as an Adjuvant Method in Combination with Chemotherapy for the Treatment of Breast Cancer in Chinese Patients: A Meta-Analysis

**DOI:** 10.1155/2018/6305645

**Published:** 2018-04-18

**Authors:** Jing-Xian Xue, Zhi-Yuan Zhu, Wei-He Bian, Chang Yao

**Affiliations:** ^1^Department of Breast Diseases, First Clinical Medical School, Nanjing University of Chinese Medicine, No. 138 Xianlin Avenue, Nanjing, Jiangsu 210023, China; ^2^Department of Breast Diseases, Jiangsu Province Hospital of Traditional Chinese Medicine/Affiliated Hospital of Nanjing University of Traditional Chinese Medicine, No. 155 Hanzhong Road, Qinhuai District, Nanjing, Jiangsu 210000, China

## Abstract

**Objective:**

The traditional Chinese medicine Kangai injection as an adjuvant method in combination with chemotherapy has been widely used for treating breast cancer in clinical practice in China. This study systematically reviewed the clinical effect and safety of traditional Chinese medicine Kangai injection as an adjuvant method in combination with chemotherapy for treating Chinese patients with breast cancer.

**Methods:**

Seven databases were searched in this study, namely, PubMed, the Cochrane Library, Embase, CNKI, Sino Med, VIP, and Wanfang Data. The timeframe of retrieval was set from the founding date of each database to November 1, 2017.

**Results:**

Fifteen papers were included in this study. The quality of all the studies included was low. All the studies were carried out among the Chinese population. Meta-analyses showed that, compared with single-use chemotherapy, using a Kangai injection combined with chemotherapy to treat Chinese breast cancer patient can increase the total effective rate [RR = 1.15, 95% CI (1.01, 1.32), and *P* = 0.033], improve the quality of life [RR = 1.30, 95% CI (1.14, 1.48), and *P* ≤ 0.001], decrease the incidence of weight loss [RR = 0.49, 95% CI (0.32, 0.77), and *P* = 0.002], decrease WBC count [RR = 0.78, 95% CI (0.68, 0.89), and *P* ≤ 0.001], decrease incidence of renal and liver dysfunction [RR = 0.58, 95% CI (0.46, 0.73), and *P* ≤ 0.001], and decrease cardiac dysfunction [RR = 0.41, 95% CI (0.18, 0.94), and *P* = 0.035]. For the incidence of gastrointestinal adverse reactions [RR = 0.89, 95% CI (0.65, 1.21), and *P* = 0.452], decreased platelet count [RR=0.49, 95% CI (0.18, 1.30), and *P* = 0.150], and alopecia [RR = 1.01, 95% CI (0.89, 1.14), and *P* = 0.879], these two groups showed no statistically significant differences.

**Conclusion:**

Kangai injection as an adjuvant method in combination with chemotherapy for treating Chinese breast cancer patients can improve their life quality and physical conditions and reduce the adverse reactions that result from chemotherapy. However, the present conclusion is only suitable for the Chinese population. The long-term, high-quality researches are required to further verify the above conclusions.

## 1. Introduction

The number of breast cancer patients has been growing in recent years. Breast cancer has become a major disease threatening women's health. Malignant tumors with high heterogeneity account for one-third of female malignant tumor patients. Over 1.5 million females in this world are newly diagnosed with breast cancer each year [1]. In 2012, GLOBOCAN evaluated 12.7 million cancer cases and 7.6 million cancer deaths and found that the incidence and mortality of breast cancer ranked number 1 among female malignant tumors [2]. China is a country with low breast cancer incidence, but its incidence has experienced a sharp rise in recent years and is particularly significant in Shanghai, Tianjin, Beijing, and other large cities [3]. It is estimated that breast cancer will take 20 years to become the malignant tumor with the highest incidence in Chinese females and the largest threat to women's health. For this reason, finding effective treatment methods is a top priority [4].

To date, there are many views on the cause and pathogenic mechanism of breast cancer, but universal agreements have not been formed. Modern medicine believes that the high-risk factors of breast cancer include family history of breast cancer, menarche, elderly primipara, nulliparity, late amenorrhea, postmenopause obesity, high level of ionizing radiation, and benign breast cancer. Its pathogenesis may relate to viral infection, nullipara or nonbreastfeeding, thyroid disease, endocrine disorder, and abnormal increase of estradiol and estrone, as well as a lack of estriol. Estradiol is considered a carcinogenic substance, and estriol can protect the body and prevent cancer. The hypofunction of immunologic surveillance in the body, dysfunction of the internal organs, and action of cancer genes lead to breast cell damage, which can develop into abnormal breast tissue proliferation and eventually become cancerous. In traditional Chinese medicine, breast cancer is classified as “Ruyan,” “Rushiyong,” “Rupi,” and “Ruhe.” Traditional Chinese medicine believes that the cause of this disease is related to emotion. Emotional disorders lead to liver Qi depression, disorders of the Qi and blood, and dysfunction of the Qi regulating the body fluid. When the body fluid is stagnant, it will form sputum; since the liver (subsumed to wood) restricts the spleen (subsumed to earth), the spleen and stomach cannot ascend lucidity and descend turbidity, leading to the generation of phlegm in the body. If there are Qi obstruction and phlegm stagnation in breast vessels for too long, a tumor will form.

Currently, the treatment for breast cancer is dominated by Western medicine, which relies more on surgery, radiotherapy, chemotherapy, hormone therapy, immunotherapy, and so on. As one of the treatments, the adverse effects of radiotherapy often cause physical damage to patients, such as upper limb swelling in the affected side, decreases in white blood cells, vomiting, and decreases in immunity, causing enormous pain to the patients. The traditional Chinese medicine Kangai injection as an adjuvant method in combination with chemotherapy has been widely applied in the clinic, and there have also been many reports on this treatment. As one of the Chinese medicines for tumor treatment, Kangai injection has been extensively used in multiple tumor treatments. This medicine is a scientifically combined Chinese medicine extract from* Astragalus* (*Astragalus membranaceus* (Fisch.) Bunge),* Ginseng* (*Panax ginseng* C. A. Mey.), and* kurorinone* (oxymatrine) by modern technology, and it is often combined with many chemotherapy drugs to treat tumors in clinical practice. (1) The major effective constituent of* Astragalus* is the dried root of the* Leguminous Plant Astragalus*, which has an antitumor effect. It is sweet in taste and warm in nature, with a meridian tropism in lung and spleen. With the highest immunological competence in* Astragalus*,* Astragalus Polysaccharide* can inhibit tumor cell proliferation and induce apoptosis. Some scholars have used* Astragalus Polysaccharide* in adjuvant chemotherapy for breast cancer and have found that* Astragalus Polysaccharide* causes fewer adverse effects, improves gastrointestinal reactions resulting from chemotherapy, and prevents the reduction of white blood cells. (2) The major effective constituents of* Ginseng* are* Ginsenoside and Ginseng Polysaccharide*, which are the major effective antitumor constituents extracted from* Ginseng*. To date, more than 80 monomer components have been separated from* Ginseng* to exert different antitumor effects via respective channels. (3)* Kushenin*, also known as oxymatrine, refers to a type of alkaloid that is extracted from the* TCM Sophora alopecuroides*. Recent studies have found that* Kushenin* can selectively kill tumor cells and inhibit tumor growth by changing the molecular sequence of the nucleic acids in cells, and such influence will be intensive and occur in many sites [5]. The mechanism of action of Kang Ai injection to resist tumor may include improvement of immune function, direct inhibition of tumor growth, and inhibition of angiogenesis [6]. However, whether this medicine can enhance the treatment effect and reduce the serious adverse reactions caused by chemotherapy is still debatable. Therefore, this article conducts meta-analyses to systematically review the safety and efficacy of Kangai injection as an adjuvant method in combination with chemotherapy for treating breast cancer and offers a theoretical basis for the integration of Chinese and Western medicine treatment for breast cancer as an evidence-based medicine.

## 2. Methods and Analysis

### 2.1. Search Strategy

Electronic network databases were searched via computer. Foreign databases included PubMed, Embase, and the Cochrane Library. Chinese databases included China National Knowledge Infrastructure (CNKI), China Biology Medicine Disc (Sino Med), the VIP information resource integration service platform (VIP), and Wanfang Data knowledge service platform (Wanfang Data). The retrieval scheme was mainly based on a combination of subject words and free words. The searched Chinese words were “Kangai zhusheye”, “Ruxianai”, “Ruxianzhongliu”, “Ruai”, “Ruxianzhongwu”, “Ruxianzhongkuai”, “Rufangzhongkuai”, “Rufangai”, “Rufangzhongliu” and “Xiongbuzhongliu”, while the searched English words were “Kangai injection”, “Kang'ai injection”, “Breast Neoplasms”, “Breast Tumors”, “Cancer of Breast”, and so on. The retrieval language was not limited, and the timeframe of the retrieval was from the founding date of each database to November 1, 2017. Nonelectronic papers and journals after 2010 were manually searched, and additional references were searched when needed to improve the recall ratio. See Supplementary Table 1 for the search strategy.

### 2.2. Inclusion and Exclusion Criteria 

#### 2.2.1. Type of Study

Randomized controlled trials (RCTs) that use Kangai injection as an adjuvant method in combination with chemotherapy to treat Chinese breast cancer patients, regardless of blinding, were used in this study. The language was also not restricted to minimize publication bias.

#### 2.2.2. Subject Investigated

(1) Diagnostic criteria were as follows: patient diagnosed by a postoperative pathology slice; (2) patient whose life quality was assessed by the Karnofsky score and heart, liver, and kidney functions were normal before treatment, with no obvious complications; and (3) studies that did not use breast cancer drugs unrelated to this study recently. The age, gender, case source, disease course, tumor classification, and chemotherapy cycle were not limited.

#### 2.2.3. Exclusion Criteria

Exclusion criteria include the following: (1) non-RCTs study; (2) inconsistent baseline information (the age, gender, case source, disease course, tumor classification, and chemotherapy cycle); (3) systematic review, important data report, and case report; the author not receiving a reply by contact article, so all data cannot be obtained; (4) therapeutic measures failing to meet the predetermined inclusion criteria; and (5) study on the type of animal test.

#### 2.2.4. Intervention

Experimental group: the chemotherapy regimen included a Kangai injection combined with conventional cytotoxic drugs [cyclophosphamide (CTX), pirarubicin (THP), fluorouracil (5-Fu), epirubicin, Adriamycin (ADM), etc. (medication dose, medication time and frequency, and treatment course)]. Control group: the chemotherapy regimen included conventional cytotoxic drugs (medication dose, medication time and frequency, and treatment course). The age, gender, and other baseline conditions of the research objects were well-matched.

#### 2.2.5. Outcome Indicators

Statistical analysis was performed on the following measures after treatment: ① total effective rate; ② improvement of the quality of life; ③ incidence of weight loss; ④ incidence of decreased WBC count; ⑤ incidence of gastrointestinal adverse reactions; ⑥ incidence of renal and liver dysfunction; ⑦ incidence of cardiac dysfunction; ⑧ incidence of decreased platelet count; and ⑨ incidence of alopecia.

The evaluation of the short-term effect was based on the Evaluation Criteria for Solid Tumor [7]. Complete remission (CR): carcinoma completely disappears for more than one month, with no recurrence or metastasis. Partial remission (PR): tumor maximum diameter *∗* maximum vertical diameter decreases by over 50% for more than one month, with no enlargement of the other lesions. Stable disease (SD): lesion maximum diameter *∗* maximum vertical diameter decreases by less than 50% or increases by less than 25% for more than one month. Progressive disease (PD): lesion maximum diameter *∗* maximum vertical diameter increases by more than 25%, or there are new lesions.

Total effective rate = (CR + PR)/(CR + PR + SD + PD)*∗*100%.

*Weight Changes*. Weight gain: weight increased after treatment more than before treatment by ≥1 kg. Weight remained stable: there is increase <1 kg. Weight loss: weight decreased after treatment more than before treatment by ≥1 kg.

*Grading Standards of WBC Count Decrease*. level 0 indicates WBC count ≥ 4.0 × 10^9^/L; level I indicates WBC count (3.0–3.9) × 10^9^/L; level II indicates WBC count (2.0–2.9) × 10^9^/L; level III indicates WBC count (1.0–1.9) × 10^9^/L; level IV indicates WBC count < 1.0 × 10^9^/L.

Quality of life was evaluated in accordance with Karnofsky Performance Status (KPS) [8]. An increase of more than 10 points in the KPS is considered an improvement, while an increase of less than 10 points in the KPS is considered stable, and a decrease of more than 10 points in the KPS is regarded as a decrease.

We use NNT (the number of patients who must be treated in order to prevent one adverse event) when RR or OR > 1 or RD < 0; we use NNH (the number of patients who need to be treated over a specific period of time before one adverse side effect of the treatment will occur) when RR or OR > 1 or RD > 0.

#### 2.2.6. Data Extraction

Two evaluators independently performed a search according to the search strategy, and preliminary screening was based on independent topics and abstracts of the search results, excluding obviously unqualified documents. A full-text methodology screening was conducted on the literature that may meet the inclusion criteria, and the authors were contacted when there was incomplete information. Then, the studies were cross-checked by two evaluators. Any disagreement on the conclusion of two evaluators was solved by a discussion. If such disagreement could not be solved through discussion, final judgment and arbitration may be made by a third party. Extracted contents included author's name, year of publication, number of samples, TNM staging, intervention, course of treatment, and observed indicators.

### 2.3. Quality Evaluation

The selected investigators simultaneously evaluated the bias risk of the included studies based on the “risk of bias” evaluation tool in* the Cochrane Handbook for Systematic Reviews of Interventions* and relevant assessment guideline regulations [9]. This risk evaluation tool contains 7 items: (1) random sequence generation; (2) allocation concealment; (3) blinding of the participants and personnel; (4) blinding of the outcome data; (5) incomplete outcome data; (6) selective reporting; and (7) other bias. The 7 items were evaluated as having a “high risk of bias,” “low risk of bias,” or “unclear risk of bias” according to the assessment criteria.

### 2.4. Data Analysis

(1) Stata 12.0 software was used to perform the statistical analysis for the meta-analyses. (2) Select effect size: if an index of the included documents is a binary variable, the curative effect analysis statistics can be represented by relative risk (RR) and expressed by its confidence interval (CI); mean difference (MD) and 95% CI were used to represent continuous changes. (3) Homogeneity test: the steps for the statistical result of the homogeneity test: test the variation degree of various original research results and clearly include the degree of homogeneity of the experiment. (4) Meta-analyses: according to the result of the heterogeneous test, *P* ≥ 0.05 and *I*^2^ < 50 indicate that the results have good agreement and that the fixed effect model (FEM) may be used. *P* < 0.05 and *I*^2^ ≥ 50 suggest that the heterogeneity of the results cannot be ignored. If the included studies still have clinical significance, the random effects model (REM) may be used. (5) Sensitivity analysis: in those meta-analyses of the comprehensive factors combined with multiple outcomes, possible anomalous studies were ruled out before reevaluation. The results were compared with those of meta-analyses before the exclusion to figure out to what extent the excluded studies would influence the combined effect size and whether those meta-analyses are stable. If there is little difference between the two results, then the sensitivity of the results is relatively low, and the results are stable, indicating high credibility. (6) Subgroup analysis: subgroup analysis was conducted on some indexes with high heterogeneity. For events in which quantitative synthesis was impossible and events with very low incidence, qualitative evaluation may be based on the description. In this study, Stata 12.0 software was used to conduct sensitivity analysis and subgroup analysis and draw a sensitivity analysis chart.

### 2.5. Publication Bias

Publication bias occurs when positive data in similar research papers with statistical significance are more likely to be published on journals. This situation is hard to control. The funnel plot method is often used to detect publication bias. Stata 12.0 software was used in this study. Egger's test was performed to detect the publication bias in the outcome measures with ≥6 included studies or *I*^2^ ≥ 30%, and a funnel plot was drawn. If a large publication bias was found in a certain research index, the exact reason should be identified. If we were unable to find the cause of the bias, the stability of the current results should be tested by the trim and filling method.

## 3. Results

### 3.1. Search Results

**Figure 1:**
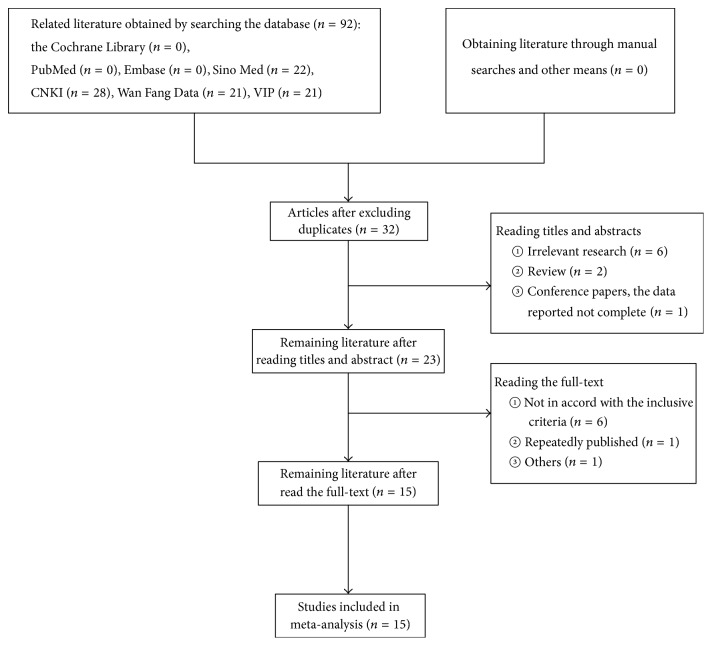
The flowchart of document selection.

A total of 92 documents [the Cochrane Library (*n* = 0), PubMed (*n* = 0), Embase (*n* = 0), Sino Med (*n* = 22), CNKI (*n* = 28), Wanfang Data (*n* = 21), and VIP (*n* = 21)] met the data collection and search strategy conditions. NoteExpress 2, a professional document management software, was employed to check for duplication of the 32 obtained articles that met the relevance requirement. After reviewing the titles and abstracts, irrelevant studies (*n* = 6), reviews (*n* = 2), and conference papers that did not provide data (*n* = 1) were eliminated. Again, documents not meeting the predetermined inclusion criteria (*n* = 6), redundant publications (*n* = 1), and other documents (*n* = 1) were ruled out by reading the full content. Finally, 15 randomized controlled trials were included (see Figure 1).

### 3.2. Study Characteristics

There were 15 randomized controlled trials (sample size: 30–236 cases) that were included in the present research [10–24] involving 1,201 patients (643 in the research group and 558 in the control group).  Table 1 showed the basic characteristics of these studies.

### 3.3. Summary of the Quality and Bias Risk of the Trials Included

**Figure 2:**
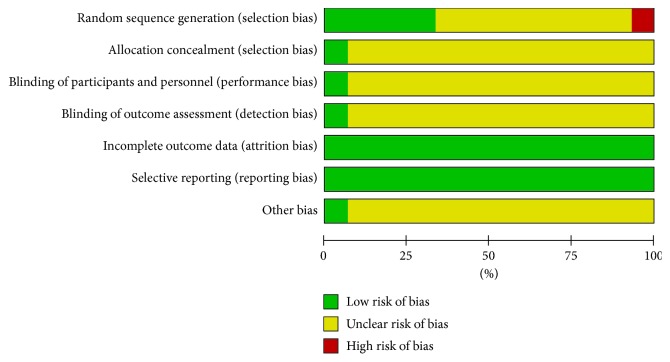
Risk of bias.

**Figure 3:**
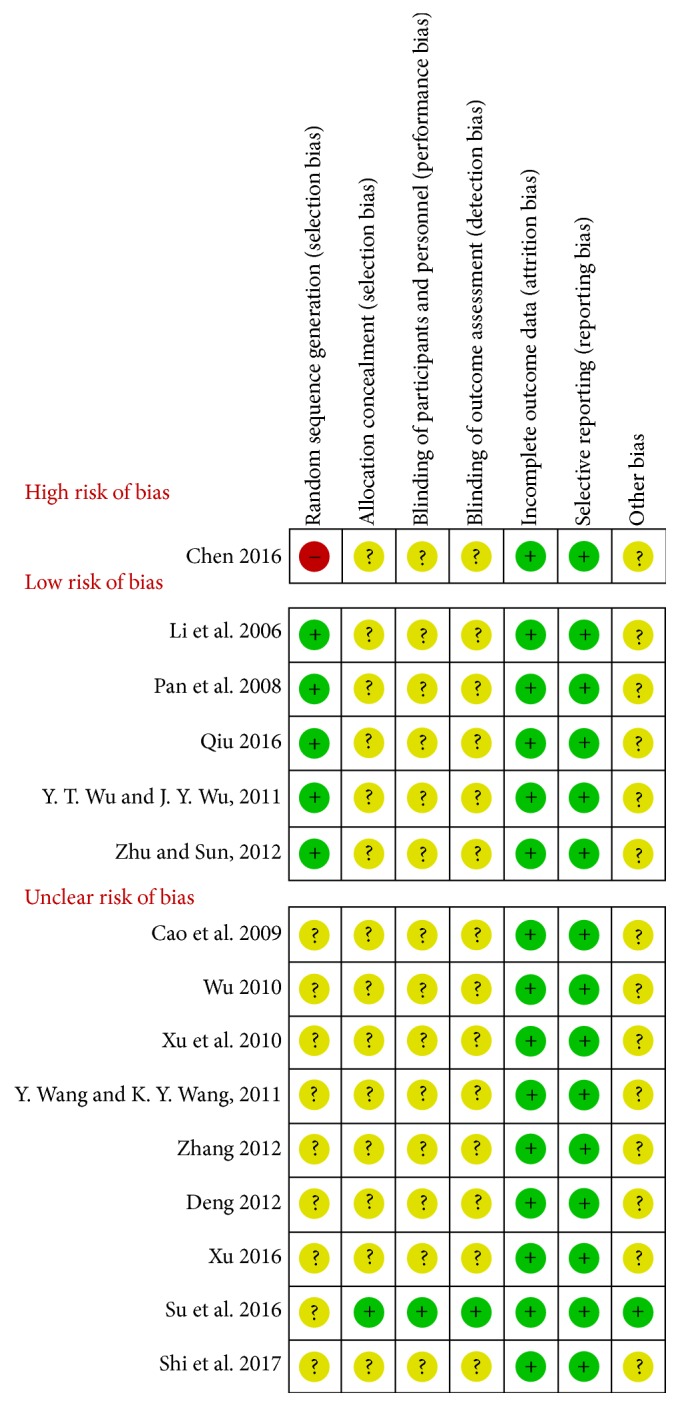
The summary of subgroup analysis for the risk of bias is based on the selection bias.

The quality of all the studies included was low. All the studies were carried out among the Chinese population. Fifteen studies [10–24] mention the use of random allocation: 5 studies [10, 16, 19, 21, 23] used the correct grouping method, 1 study [20] employed the wrong grouping method, and the rest of the studies failed to mention the specific grouping method. Only 1 study [22] discussed allocation concealment, blinding, and evaluator blinding. The quality assessment was shown in Figures 2 and 3.

### 3.4. Outcome Measures

#### 3.4.1. Total Effective Rate

**Figure 4:**
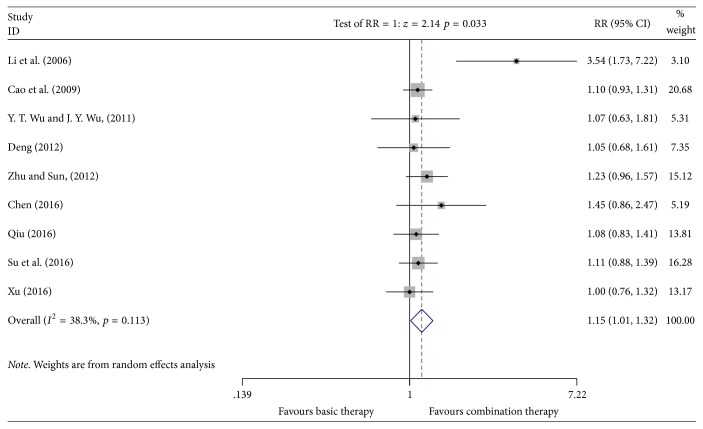
Meta-analyses results of Kangai injection combined with chemotherapy versus chemotherapy alone in terms of the total effective rate for breast cancer.

A total of 9 studies [10, 12, 16, 17, 19–23] reported the results of the total effective rate, involving 854 patients (the experimental group included 466 patients; the control group included 388 patients). The heterogeneity test showed no heterogeneity (*P* = 0.113, *I*^2^ = 38.3%). The random effects model was used for the data analysis. Meta-analyses indicated that the total clinical effective rate of Kangai injection combined with chemotherapy in treating breast cancer was higher than that in the control group, which showed a statistically significant difference [*n* = 9, RR = 1.15, 95% CI (1.01, 1.32), and *P* = 0.033] [RD = 0.12 > 0, CI (0.03, 0.21), and NNH = 8] (see Figure 4). The results of the sensitivity analysis could be found in Supplementary Figure S1.

#### 3.4.2. Improvement of the Quality of Life

**Figure 5:**
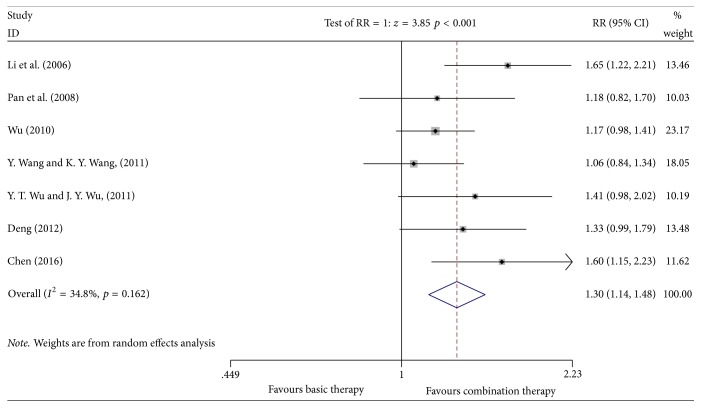
Meta-analyses results of Kangai injection combined with chemotherapy versus chemotherapy alone in terms of the improvement of quality of life for breast cancer.

Seven studies [10, 11, 13, 15–17, 20] reported the improvement of the quality of life, involving 438 patients (the experimental group included 221 patients; the control group included 217 patients). A heterogeneity test showed heterogeneity in these studies (*P* = 0.162, *I*^2^ = 34.8%). The random effects model was used for data analysis. Meta-analyses suggested that the improvement of the quality of life of the Kangai injection combined with chemotherapy in treating breast cancer was higher than that in the control group, which showed a statistically significant difference [*n* = 7, RR = 1.30, 95% CI (1.14, 1.48), and *P* ≤ 0.001] [RD = 0.21 > 0, CI (0.12, 0.30), and NNH = 5] (see Figure 5). Supplementary Figure S2 showed the results of the sensitivity analysis.

#### 3.4.3. Incidence of Weight Loss

**Figure 6:**
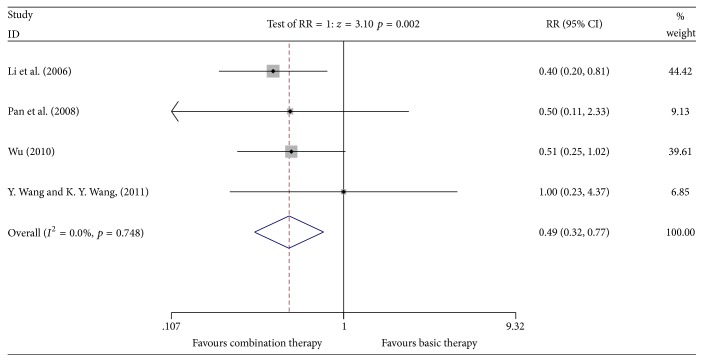
Meta-analyses results of Kangai injection combined with chemotherapy versus chemotherapy alone in terms of the incidence of weight loss for breast cancer.

Four studies [10, 11, 13, 15] reported the incidence of weight loss, involving 248 patients (the experimental group included 126 patients; the control group included 122 patients). A heterogeneity test showed no heterogeneity in these studies (*P* = 0.748, *I*^2^ = 0.0%). The fixed effects model was used for the data analysis. Meta-analyses suggested that the incidence of weight loss in the Kangai injection combined with chemotherapy for treating breast cancer was lower than in the control group, which showed a statistically significant difference [*n* = 4, RR = 0.49, 95% CI (0.32, 0.77), and *P* = 0.002] [RD = −0.18 < 0, CI (−0.28, −0.07), and NNT = 6] (see Figure 6).

#### 3.4.4. Incidence of WBC Count Decrease

**Figure 7:**
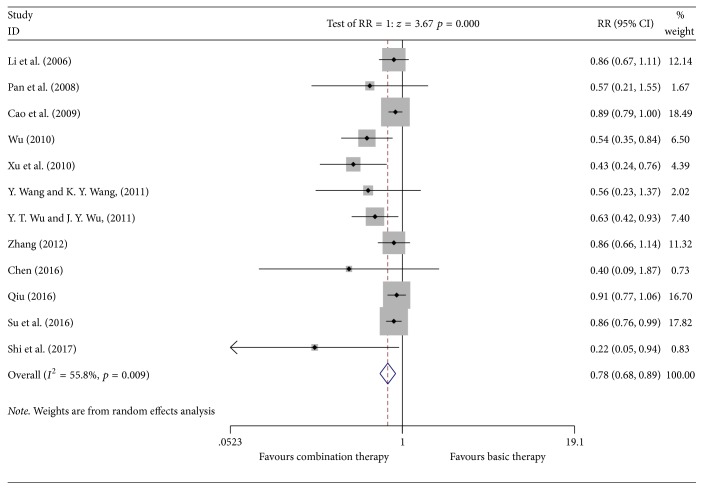
Meta-analyses results of Kangai injection combined with chemotherapy versus chemotherapy alone in terms of the incidence of decreased WBC count for breast cancer.

Twelve studies [10–16, 18, 20–22, 24] reported the incidence of decreased WBC count involving 1033 patients (the experimental group included 560 patients; the control group included 473 patients). A heterogeneity test showed heterogeneity (*P* = 0.009, *I*^2^ = 55.8%). A random effects model was used for data analysis. Meta-analyses suggested that the incidence of decreased WBC count in Kangai injection combined with chemotherapy in treating breast cancer was lower than that in the control group, which showed a statistically significant difference [*n* = 12, RR = 0.78, 95% CI (0.68, 0.89), and *P* ≤ 0.001] [RD = −0.16 < 0, CI (−0.21, −0.10), and NNT = 6] (see Figure 7). Supplementary Figure S3 showed the result of the sensitivity analysis.

#### 3.4.5. Incidence of Gastrointestinal Adverse Reactions

**Figure 8:**
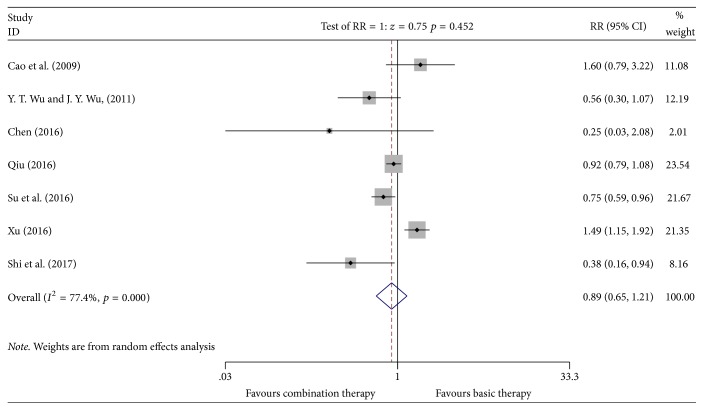
Meta-analyses results of Kangai injection combined with chemotherapy versus chemotherapy alone in terms of incidence of gastrointestinal adverse reactions for breast cancer.

Seven studies [12, 16, 20–24] reported the incidence of gastrointestinal adverse reactions involving 692 patients (the experimental group included 384 patients; the control group included 308 patients). A heterogeneity test showed heterogeneity (*P* ≤ 0.001, *I*^2^ = 77.4%). The random effects model was used for data analysis. Meta-analyses suggested that for the incidence of gastrointestinal adverse reactions in Kangai injection combined with chemotherapy for treating breast cancer there was no significant difference between the two groups [*n* = 7, RR = 0.89, 95% CI (0.65, 1.21), and *P* = 0.452] [RD = −0.06 < 0, CI (−0.20, −0.08), and NNT = 17] (see Figure 8). Supplementary Figure S4 showed the results of the sensitivity analysis.

#### 3.4.6. Incidence of Renal and Liver Dysfunction

**Figure 9:**
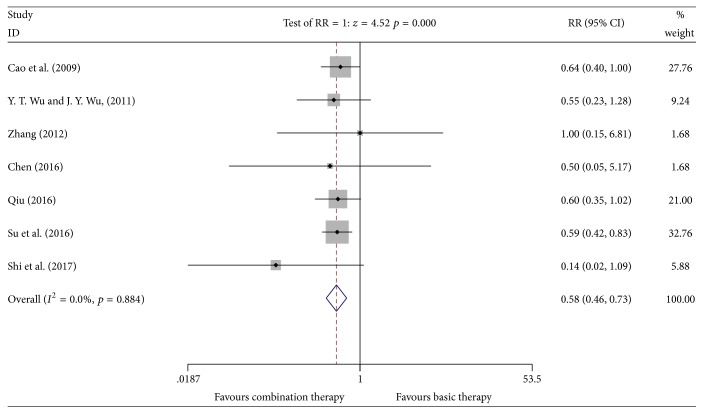
Meta-analyses results of Kangai injection combined with chemotherapy versus chemotherapy alone in terms of the incidence of renal and liver dysfunction for breast cancer.

Seven studies [12, 16, 18, 20–22, 24] reported the incidence of renal and liver dysfunction involving 728 patients (the experimental group included 402 patients; the control group included 326 patients). A heterogeneity test showed no heterogeneity (*P* = 0.884, *I*^2^ = 0.0%). The fixed effects model was used for data analysis. Meta-analyses suggested that the incidence of renal and liver dysfunction of the Kangai injection combined with chemotherapy in treating breast cancer was lower than that in the control group, which showed no statistically significant difference [*n* = 7, RR = 0.58, 95% CI (0.46, 0.73), and *P* ≤ 0.001] [RD = −0.14 < 0, CI (−0.20, −0.08), and NNT = 7] (see Figure 9).

#### 3.4.7. Incidence of Cardiac Dysfunction

**Figure 10:**
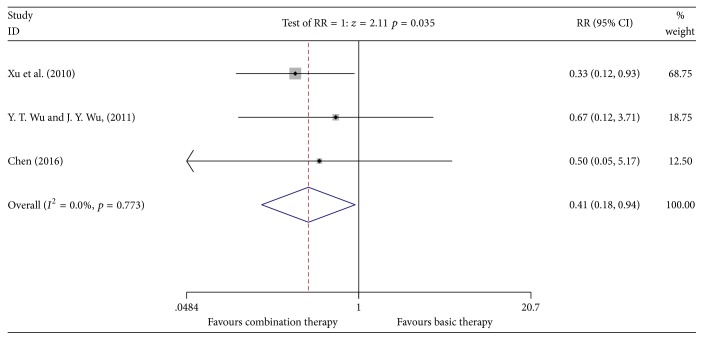
Meta-analyses results of Kangai injection combined with chemotherapy versus chemotherapy alone in terms of incidence of cardiac dysfunction for breast cancer.

Three studies [14, 16, 20] reported the incidence of cardiac dysfunction involving 162 patients (the experimental group included 88 patients; the control group included 82 patients). A heterogeneity test showed no heterogeneity (*P* = 0.773, *I*^2^ = 0.0%). The fixed effects model was used for data analysis. Meta-analyses suggested that the incidence of cardiac dysfunction of the Kangai injection combined with chemotherapy in treating breast cancer was lower than that in the control group, which showed statistically significant difference [*n* = 3, RR = 0.41, 95% CI (0.18, 0.94), and *P* = 0.035] [RD = −0.11 < 0, CI (−0.21, −0.01), and NNT = 9] (see Figure 10).

#### 3.4.8. Incidence of Decreased Platelet Count

**Figure 11:**
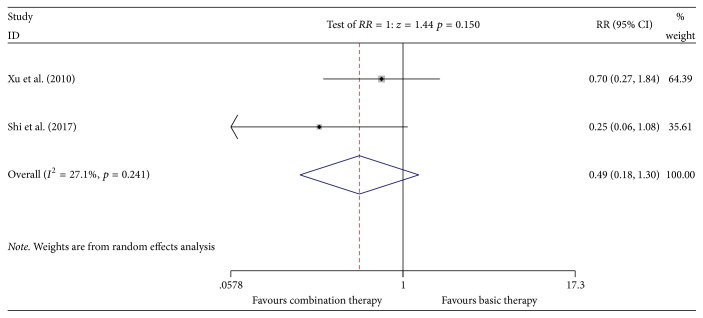
Meta-analyses results of Kangai injection combined with chemotherapy versus chemotherapy alone in terms of incidence of decreased platelet count for breast cancer.

Two studies [14, 24] reported the incidence of decreased platelet count involving 120 patients (the experimental group included 63 patients; the control group included 57 patients). A heterogeneity test showed low heterogeneity (*P* = 0.241, *I*^2^ = 27.1%). The random effects model was used for data analysis. Meta-analyses suggested that, for the incidence of decreased platelet count in the Kangai injection combined with chemotherapy for treating breast cancer, there was no significant difference between the two groups [*n* = 2, RR = 0.49, 95% CI (0.18, 1.30), and *P* = 0.150] [RD = −0.15 < 0, CI (−0.29, −0.01), and NNT = 7] (see Figure 11).

#### 3.4.9. Incidence of Alopecia

**Figure 12:**
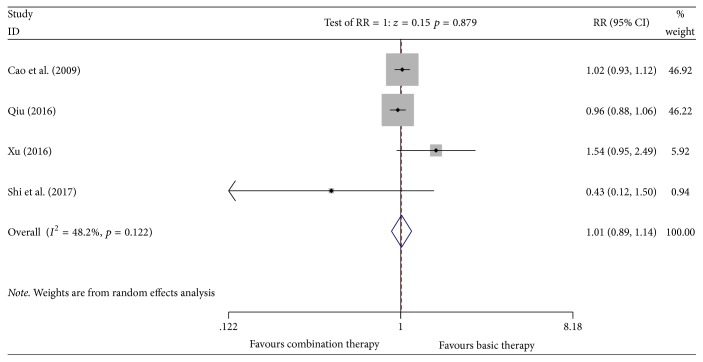
Meta-analyses results of Kangai injection combined with chemotherapy versus chemotherapy alone in terms of incidence of alopecia for breast cancer.

Four studies [12, 21, 23, 24] reported the incidence of alopecia involving 476 patients (the experimental group included 276 patients; the control group included 200 patients). A heterogeneity test showed heterogeneity (*P* = 0.122, *I*^2^ = 48.2%). The random effect model was used for data analysis. Meta-analyses suggested that, for the incidence of alopecia in the Kangai injection combined with chemotherapy for treating breast cancer, there was no significant difference between the two groups [*n* = 4, RR = 1.01, 95% CI (0.89, 1.14), and *P* = 0.879] [RD = −0.01 < 0, CI (−0.10, −0.09), and NNT = 100] (see Figure 12).

### 3.5. Publication Bias

*Publication Bias*. Stata 12.0 software was used to check the publication bias for the measures with *I*^2^ ≥ 30 and the number of studies ≥ 6 among the outcome measures. *P* ≤ 0.05 suggested publication bias. Egger's test was carried out. If any large publication bias was found, the trim and fill method would be employed to check the stability of the result.

*Total Effective Rate*. Egger's test indicated no publication bias (*P* = 0.112, 95% CI (−0.5122263, 3.915406)). The specific results are showed in Supplementary Figure S5.

*Improvement of the Quality of Life*. Egger's test found no publication bias (*P* = 0.162, 95% CI (−1.515151, 6.831656)). The specific results are showed in Supplementary Figure S6.

*Incidence of Decreased WBC Count*. Egger's test suggested publication bias (*P* ≤ 0.001, 95% CI (−2.446743, 0.9831339)). The result of the trim and fill method with Stata 12.0 software showed that the data was unchanged. The specific results are showed in Supplementary Figure S7.

*Incidence of Gastrointestinal Adverse Reactions*. Egger's test revealed no publication bias (*P* = 0.553, 95% CI (−4.422824, 2.670058)). The specific results could be found in Supplementary Figure S8.

The research results above suggested that the heterogeneity of the incidence of decreased leukocyte count was high, and there might be certain publication biases. This may be associated with the small quantity of included studies.

## 4. Discussion

Breast cancer is the most common tumor among modern females. Its incidence across the world in on the rise, ranking number 1 in all female malignant tumors in Western countries. At present, major breast cancer treatment involves chemotherapy and radiotherapy following surgical treatment. Chemotherapy is the most active and effective therapeutic measure during various periods of treating breast cancer [25]. However, the adverse effects caused by chemotherapy are also cumbersome. As a result, reducing these adverse effects and improving the patient's quality of life are particularly important [26].

The occurrence and development of breast cancer are the result of the interactions among multiple factors and multiple mechanisms. A single therapeutic approach cannot address this complicated problem. As a traditional treatment means for breast cancer, traditional Chinese medicine (TCM) treatment is a promising approach for the research and treatment of breast cancer. In recent years, TCM treatment has become part of modern comprehensive therapy. More and more reports have proven its unique effect and its role in compensating for deficiencies in Western medicine treatment. Both the internal treatment and external treatment of TCM show good effects in treating postoperative complications, promoting postoperative recovery, enhancing the body's immunity, reducing chemotherapy and radiotherapy toxicity, and improving efficacy. Traditional Chinese medicine tumor treatment involves such methods as strengthening healthy Qi to eliminate pathogens, clearing heat and removing toxicity, promoting blood circulation to remove blood stasis, dispelling phlegm and eliminating dampness, and softening hardness to dissipate stagnation [27]. In the present research, the selected TCM, the Kangai injection, is a TCM compound that aims to treat the tumor by strengthening the healthy Qi to eliminate pathogens under the guidance of the basic theory of traditional Chinese medicine. It has been widely used to treat various tumors of different types in China as an adjuvant treatment. Therefore, this research systematically evaluates the clinical effectiveness and safety of the TCM Kangai injection as an adjuvant means in combination with chemotherapy for treating Chinese breast cancer patients, thus providing theoretical evidence, in terms of evidence-based medicine, for the integration of Chinese and Western medicine in the treatment of breast cancer.

The results of the present research indicated that, compared with chemotherapy alone, Kangai injection as an adjuvant method in combination with chemotherapy could increase the total effective rate and improve the quality of life, as well as decreasing the incidence of weight loss, decreased WBC count, liver and renal dysfunction, and abnormal cardiac dysfunction; however, there were no statistically significant differences between the two therapeutic methods in the incidence of gastrointestinal adverse reactions, decreased platelet count, and alopecia. The immune system related indicators of the patients in the combined treatment group were significantly improved after the treatment. Description of Kangai injection had played a positive role in improving the immune function of patients with tumor. The patients' immune function was improved and the patients' ability to resist the adverse effects of chemotherapy was also enhanced [28]. In a word, Kangai injection was effective in the treatment of breast cancer.

## 5. Limitations and Advantages

Multidrug resistance of tumor cells is a major barrier in tumor chemotherapy and can promote tumor cells to generate resistance to various structures and types of drugs with different mechanisms of action, thus compromising the antitumor effect and leading to chemotherapy failure. The major effect of TCM in tumor treatment is not achieved by acting on only one target. Instead, such an effect is realized by strengthening the immune function [28], downregulating the expression of vascular endothelial growth factor (VEGF), regulating the expression of apoptosis-related genes, reversing multidrug resistance of the tumor cells, or directly killing the tumor cells. The effective constituents of many traditional Chinese medicines demonstrate good effects for inhibiting tumor cell proliferation and inducing differentiation with fewer or even no adverse reactions. The limitation of this research primarily lies in publication bias [29, 30]. The possible reasons are as follows: (1) Most of the clinical studies included in this research were carried out in China, and all the subjects were Chinese people. The current findings fail to prove that this combination therapy also has the same effect among other populations. (2) All the documents obtained are written in Chinese, and most of them do not report allocation concealment, blinding, and specific allocation principles. Consequently, the quality of the documents included is relatively low. Additionally, these documents lack further follow-ups and fail to study the relapse rate and total survival time. Thus, the long-term effect of this combination therapy in this research remains unclear. (3) Researchers and periodical offices prefer to publish clinical research papers with positive results, leading to publication bias due to the lack of documents with negative results and thus resulting in an overestimation of the real therapeutic effect.

## 6. Conclusion

Kangai injection as an adjuvant method in combination with chemotherapy for treating Chinese breast cancer patients can improve their life quality and physical conditions and reduce the adverse reactions that result from chemotherapy. However, the present conclusion is only suitable for the Chinese population. The long-term, high-quality researches with a large sample size in different populations are required to further verify the above conclusions.

## Figures and Tables

**Table 1:** The basic characteristics of the 15 included studies.

Author/year	Groups	Sample size	Age (median or mean or range) (year)	Is the baseline consistent	Course of treatment (days)	Intervention	Treatment options	Outcomes
Li et al. 2006 [10]	EG	42	58	Yes	21 days, 4 periods	KAI: 40 ml, ivgtt, 1 time a day, for 21 days + CTF.	CTF	①②③④
	CG	40	60		21 days, 4 periods	CTF (CTX: 500 mg/m^2^, ivgtt, the first and eighth day;		
						THP: 40 mg/m^2^, ivgtt, the first day;		
						5-Fu: 500 mg/m^2^, ivgtt, the first and eighth day).		

Pan et al. 2008 [11]	EG	15	56.62	Yes	21 days, 4 periods	KAI: 60 ml, ivgtt, 1 time a day, for 21 days + CEF.	CEF	②③④
	CG	15	59.74		21 days, 4 periods	CEF (CTX: 500 mg/m^2^, ivgtt, the first and eighth day; EPI: 60 mg/m^2^, ivgtt, the first day;		
						5-Fu: 750 mg/m^2^, ivgtt, the first and eighth day).		

Cao et al. 2009 [12]	EG	156	45	Yes	21 days, 4 periods	KAI: 60 ml, ivgtt, 1 time a day, for 21 days + CEF.	CEF	①④⑥⑦⑨
	CG	80	45		21 days, 4 periods	CEF (CTX: 500 mg/m^2^, ivgtt, the first and eighth day; EPI: 60 mg/m^2^, ivgtt, the first day;		
						5-Fu: 750 mg/m^2^, ivgtt, the first and eighth day).		

Wu 2010 [13]	EG	49	52.2 ± 11.2	Yes	21 days, 4 periods	KAI: 60 ml, ivgtt, 1 time a day, for 21 days + CEF.	CEF	②③④
	CG	47	50.7 ± 10.5		21 days, 4 periods	CEF (CTX: 500 mg/m^2^, ivgtt, the first and eighth day; EPI: 50 mg/m^2^, ivgtt, the first day;		
						5-Fu: 500 mg/m^2^, ivgtt, the first and eighth day).		

Xu et al. 2010 [14]	EG	33	54.6	Yes	21 days, 4 periods	KAI: 60 ml, ivgtt, 1 time a day, for 21 days + CEF.	CEF	④⑤⑧
	CG	27	53.5		21 days, 4 periods	CEF (CTX: 600 mg/m^2^, ivgtt, the first day; EPI: 100 mg/m^2^, ivgtt, the first day;		
						5-Fu: 500 mg/m^2^, ivgtt, the first day).		

Y. Wang and K. Y. Wang 2011 [15]	EG	20	46	Yes	21 days, 2 periods	KAI: 40 ml, ivgtt, 1 time a day, for 42 days + CTF.	CTF	②③④
	CG	20	46		21 days, 2 periods	CTF (CTX: 500 mg/m^2^, ivgtt, the first and eighth day;		
						THP: 40 mg/m^2^, ivgtt, the first day;		
						5-Fu: 500 mg/m^2^, ivgtt, the first and eighth day).		

Y. T. Wu and J. Y. Wu 2011 [16]	EG	30	51.5	Yes	21 days, 2 periods	KAI: 20 ml, ivgtt, 1 time a day, for 42 days + CTF.	CTF	①②④⑥⑦⑧
	CG	30	50.5		21 days, 2 periods	CTF (CTX: 500 mg/m^2^, ivgtt, the first day;		
						THP: 40 mg/m^2^, ivgtt, the first day;		
						5-Fu: 500 mg/m^2^, ivgtt, the first day).		

Deng 2012 [17]	EG	40	45.58	Yes	21 days, 2 periods	KAI: 60 ml, ivgtt, 1 time a day, for 42 days + CTF.	CTF	①②
	CG	40	43.63		21 days, 2 periods	CTF (CTX: 500 mg/m^2^, ivgtt, the first and eighth day;		
						THP: 40 mg/m^2^, ivgtt, the first day;		
						5-Fu: 500 mg/m^2^, ivgtt, the first and eighth day).		

Zhang 2012 [18]	EG	30	32~69	Yes	30 days, 2 periods	KAI: 40–60 ml, ivgtt, 1 time a day, for 30 days + AC.	AC	⑥
	CG	31	34~68		30 days, 2 periods	AC (CTX: 600 mg/m^2^, ivgtt, the first day;		
						ADM: 60 mg/m^2^, ivgtt, the first day)		

Zhu and Sun 2012 [19]	EG	30	52 ± 5	Yes	21 days, 4 periods	KAI: 40–60 ml, ivgtt, 1 time a day, for 30 days + CEF.	CEF	①
	CG	30	54 ± 3		21 days, 4 periods	CEF (CTX: 500 mg/m^2^, ivgtt, the first and eighth day;		
						EPI: 50 mg/m^2^, ivgtt, the first day;		
						5-Fu: 500 mg/m^2^, ivgtt, the first and eighth day).		

Chen 2016 [20]	EG	25	47.52 ± 10.31	Yes	21 days, 2 periods	KAI: 60 ml, ivgtt, 1 time a day, for 42 days + CEF.	CEF	①②④⑥⑦⑧
	CG	25	48.06 ± 10.28		21 days, 2 periods	CEF (CTX: 500 mg/m^2^, ivgtt, the first and eighth day;		
						EPI: 60 mg/m^2^, ivgtt, the first day;		
						5-Fu: 750 mg/m^2^, ivgtt, the first and eighth day).		

Qiu 2016 [21]	EG	60	45.8 ± 10.3	Yes	21 days, 4 periods	KAI: 40 ml, ivgtt, 1 time a day, for 84 days + CEF.	CEF	①④⑥⑦⑨
	CG	60	46.7 ± 10.8		21 days, 4 periods	CEF (CTX: 500 mg/m^2^, ivgtt, the first day;		
						EPI: 80–100 mg/m^2^, ivgtt, the first day;		
						5-Fu: 500 mg/m^2^, ivgtt, the first day).		

Su et al. 2016 [22]	EG	53	35~70	Yes	28 days, 3 periods	KAI: 60 ml, ivgtt, 1 time a day, for 84 days + CTF.	CTF	①④⑥⑦
	CG	53	35~70		28 days, 3 periods	CTF (CTX: 500 mg/m^2^, ivgtt, the first and fifth day;		
						THP: 40 mg/m^2^, ivgtt, the first day;		
						5-Fu: 500 mg/m^2^, ivgtt, the first and fifth day).		

Xu 2016 [23]	EG	30	42.61 ± 2.13	Yes	21 days, 4 periods	KAI: 40 ml, ivgtt, 1 time a day, for 84 days + CEF.	CEF	①⑥⑨
	CG	30	42.29 ± 2.31		21 days, 4 periods	CEF (CTX: 600 mg/m^2^, ivgtt, the first day;		
						EPI: 80–100 mg/m^2^, ivgtt, the first day;		
						5-Fu: 500 mg/m^2^, ivgtt, the first day).		

Shi et al. 2017 [24]	EG	30	41.83 ± 4.31	Yes	21 days, 4 periods	KAI: 40 ml, ivgtt, 1 time a day, for 63 days + CAF.	CAF	④⑤⑥⑦⑨
	CG	30	42.04 ± 4.25		21 days, 4 periods	CAF (CTX: 500 mg/m^2^, ivgtt, the first day;		
						ADM: 50 mg/m^2^, ivgtt, the first day;		
						5-Fu: 500 mg/m^2^, ivgtt, the first day).		

① Total effective rate; ② improvement of quality of life; ③ incidence of weight loss; ④ incidence of WBC count decrease; ⑤ incidence of decreased platelet count; ⑥ incidence of gastrointestinal adverse reactions; ⑦ incidence of renal and liver dysfunction; ⑧ incidence of cardiac dysfunction; ⑨ incidence of alopecia. EG: experimental group; CG: control group. Cyclophosphamide: CTX. THP. Fluorouracil: 5-Fu. Intravenous drip: ivgtt. Kangai injection: KAI. EPI. Adriamycin: ADM.

## Supplementary Materials

Supplementary 1 Supplementary Figure S1: the sensitivity analysis results of the total effective rate (PDF).

Supplementary 2 Supplementary Figure  S2: the sensitivity analysis results of the improvement of the quality of life (PDF).

Supplementary 3 Supplementary Figure  S3: the sensitivity analysis results of the incidence of decreased WBC count (PDF).

Supplementary 4 Supplementary Figure  S4: the sensitivity analysis results of the incidence of gastrointestinal adverse reactions (PDF).

Supplementary 5 Supplementary Figure  S5: Egger's publication bias plot of the total effective rate (PDF).

Supplementary 6 Supplementary Figure  S6: Egger's publication bias plot of the improvement of the quality of life (PDF).

Supplementary 7 Supplementary Figure  S7: Egger's publication bias plot of the incidence of decreased WBC count (PDF).

Supplementary 8 Supplementary Figure S8: Egger's publication bias plot of time of the incidence of gastrointestinal adverse reactions (PDF).

Supplementary 9 Supplementary Table 1: search terms (PDF).

Supplementary 10 Supplementary Table 2: the publication bias results of disappearance of total effective rate with Egger's test (DOCX).

Supplementary 11 Supplementary Table 3: the publication bias results of disappearance of improvement of quality of life with Egger's test (DOCX).

Supplementary 12 Supplementary Table 4: the publication bias results of disappearance of incidence of WBC count decrease with Egger's test (DOCX).

Supplementary 13 Supplementary Table 5: the publication bias results of disappearance of incidence of gastrointestinal adverse reactions with Egger's test (DOCX).
