# Supplementary material for: The Traditional Chinese Medicine Kangai Injection as an Adjuvant Method in Combination with Chemotherapy for the Treatment of Breast Cancer in Chinese Patients: A Meta-Analysis
Source: Evid Based Complement Alternat Med. 2018 Apr 18;2018:6305645. doi: 10.1155/2018/6305645 (PMC5932437; doi:10.1155/2018/6305645)
Supplement: Supplementary 2 — Supplementary Figure S2: the sensitivity analysis results of the improvement of the quality of life (PDF). [file 6305645.f2.pdf]

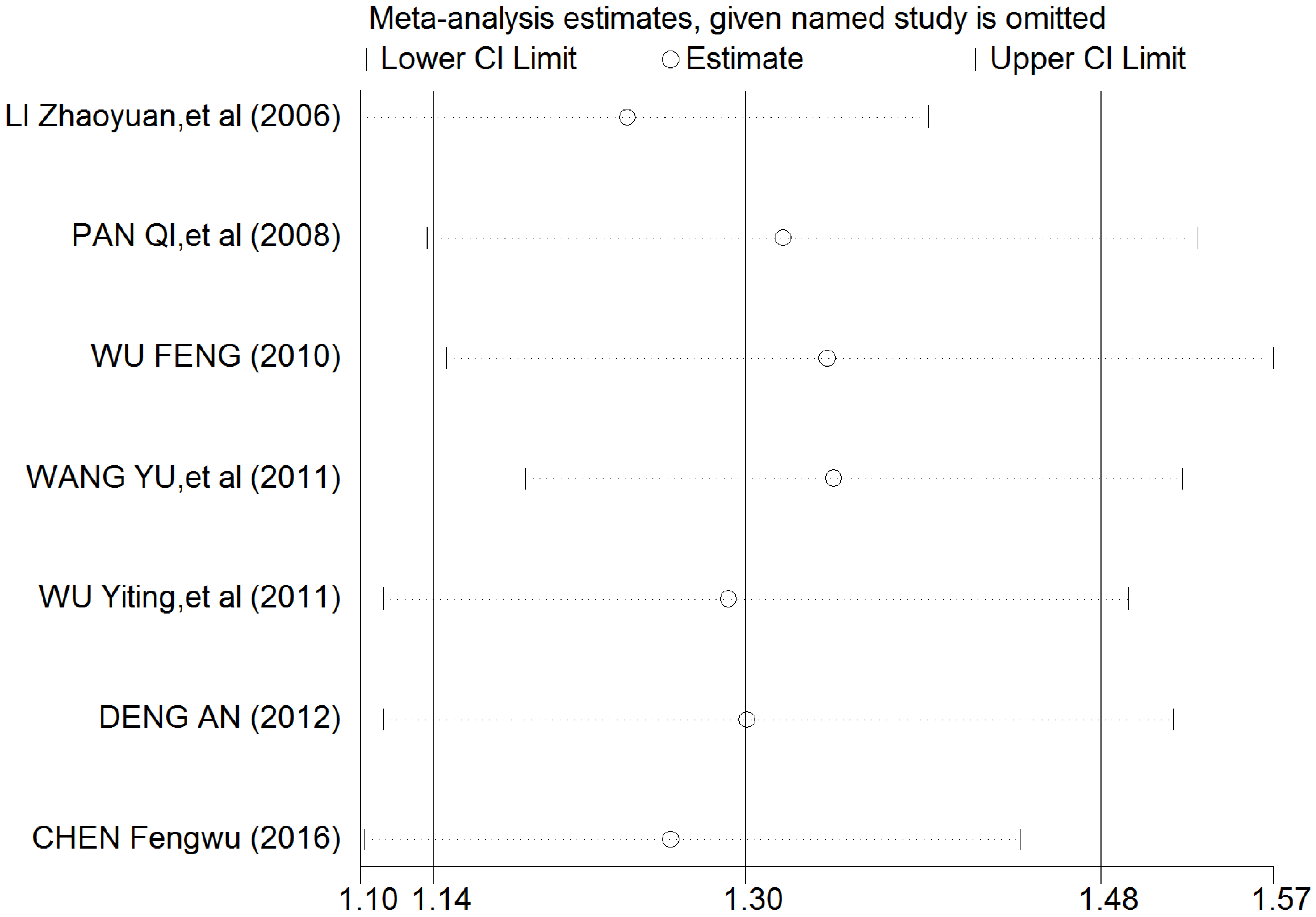

Meta-analysis estimates, given named study is omitted
Lower CI Limit
Estimate
Upper CI Limit
LI Zhaoyuan,et al (2006)
PAN QI,et al (2008)
WU FENG (2010)
WANG YU,et al (2011)
WU Yiting,et al (2011)
DENG AN (2012)
CHEN Fengwu (2016)
1.10
1.14
1.30
1.48
1.57
