# Supplementary material for: The Traditional Chinese Medicine Kangai Injection as an Adjuvant Method in Combination with Chemotherapy for the Treatment of Breast Cancer in Chinese Patients: A Meta-Analysis
Source: Evid Based Complement Alternat Med. 2018 Apr 18;2018:6305645. doi: 10.1155/2018/6305645 (PMC5932437; doi:10.1155/2018/6305645)
Supplement: Supplementary 3 — Supplementary Figure S3: the sensitivity analysis results of the incidence of decreased WBC count (PDF). [file 6305645.f3.pdf]

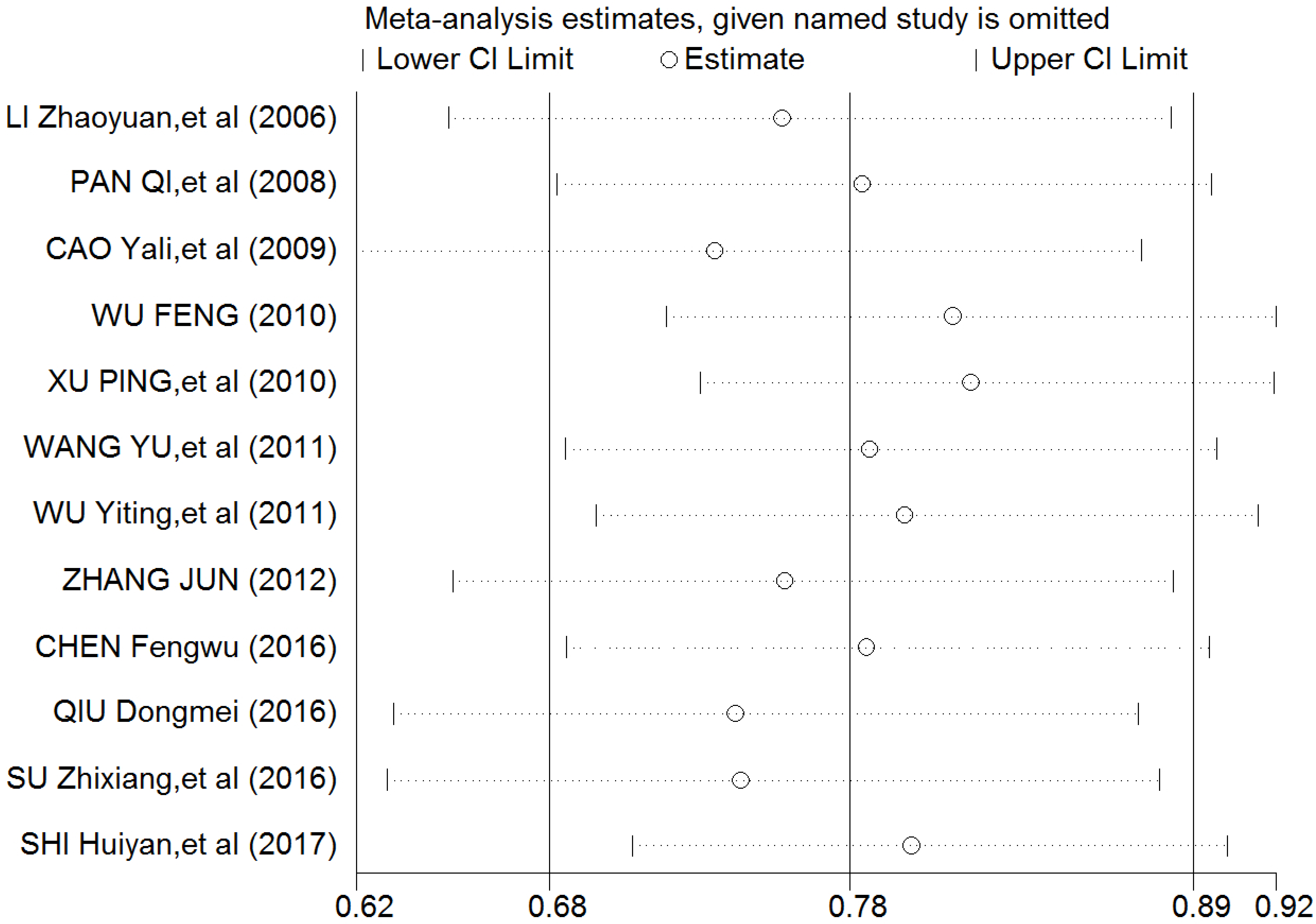
Meta-analysis estimates, given named study is omitted
Lower CI Limit
Estimate
Upper CI Limit
LI Zhaoyuan,et al (2006)
PAN QI,et al (2008)
CAO Yali,et al (2009)
WU FENG (2010)
XU PING,et al (2010)
WANG YU,et al (2011)
WU Yiting,et al (2011)
ZHANG JUN (2012)
CHEN Fengwu (2016)
QIU Dongmei (2016)
SU Zhixiang,et al (2016)
SHI Huiyan,et al (2017)
0.62
0.68
0.78
0.89
0.92
