# Supplementary figures and images for: The Traditional Chinese Medicine Kangai Injection as an Adjuvant Method in Combination with Chemotherapy for the Treatment of Breast Cancer in Chinese Patients: A Meta-Analysis
Source: Evid Based Complement Alternat Med. 2018 Apr 18;2018:6305645. doi: 10.1155/2018/6305645 (PMC5932437; doi:10.1155/2018/6305645)

Meta-analysis estimates, given named study is omitted

| Lower CI Limit

○ Estimate

| Upper CI Limit

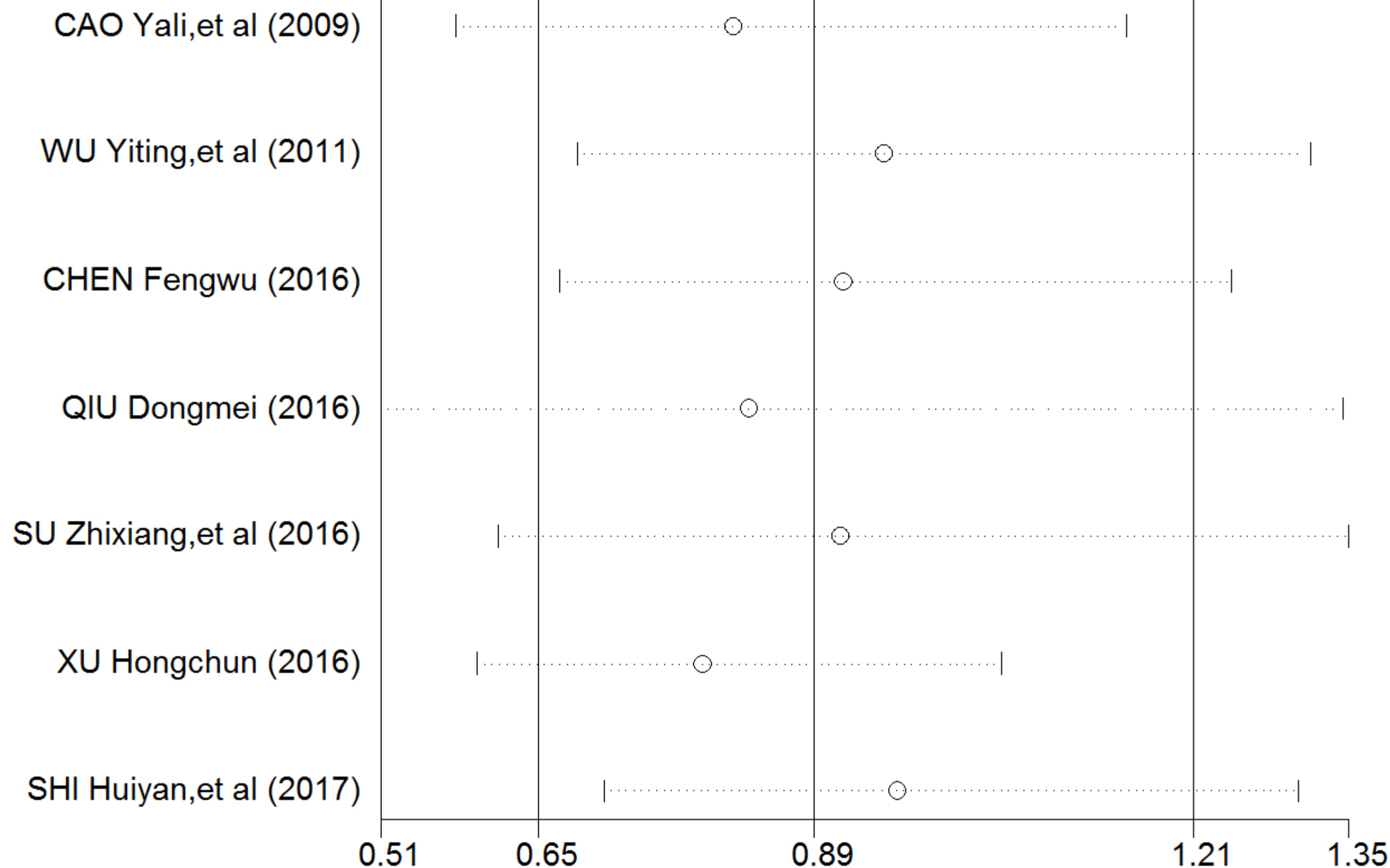

Supplement: Supplementary 4 — Supplementary Figure S4: the sensitivity analysis results of the incidence of gastrointestinal adverse reactions (PDF). [file 6305645.f4.pdf]

Egger's publication bias plot

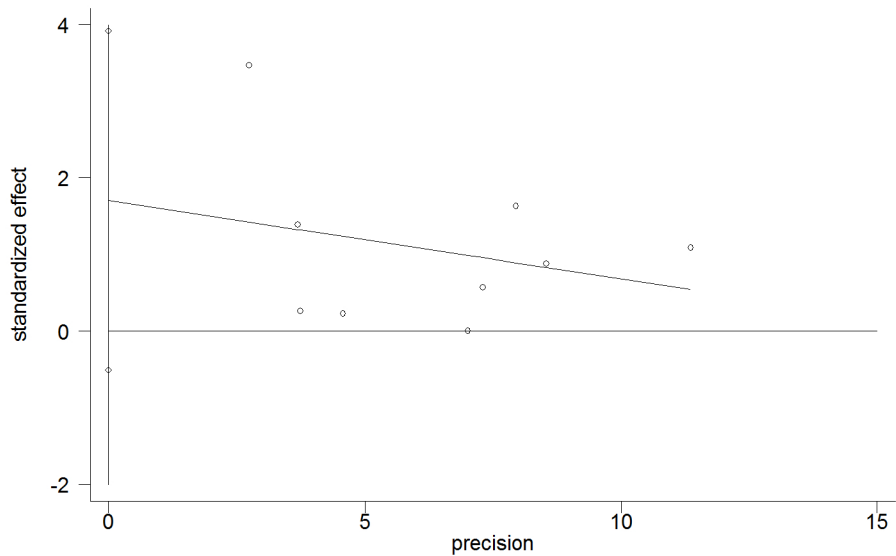

Supplement: Supplementary 5 — Supplementary Figure S5: Egger's publication bias plot of the total effective rate (PDF). [file 6305645.f5.pdf]

Egger's publication bias plot

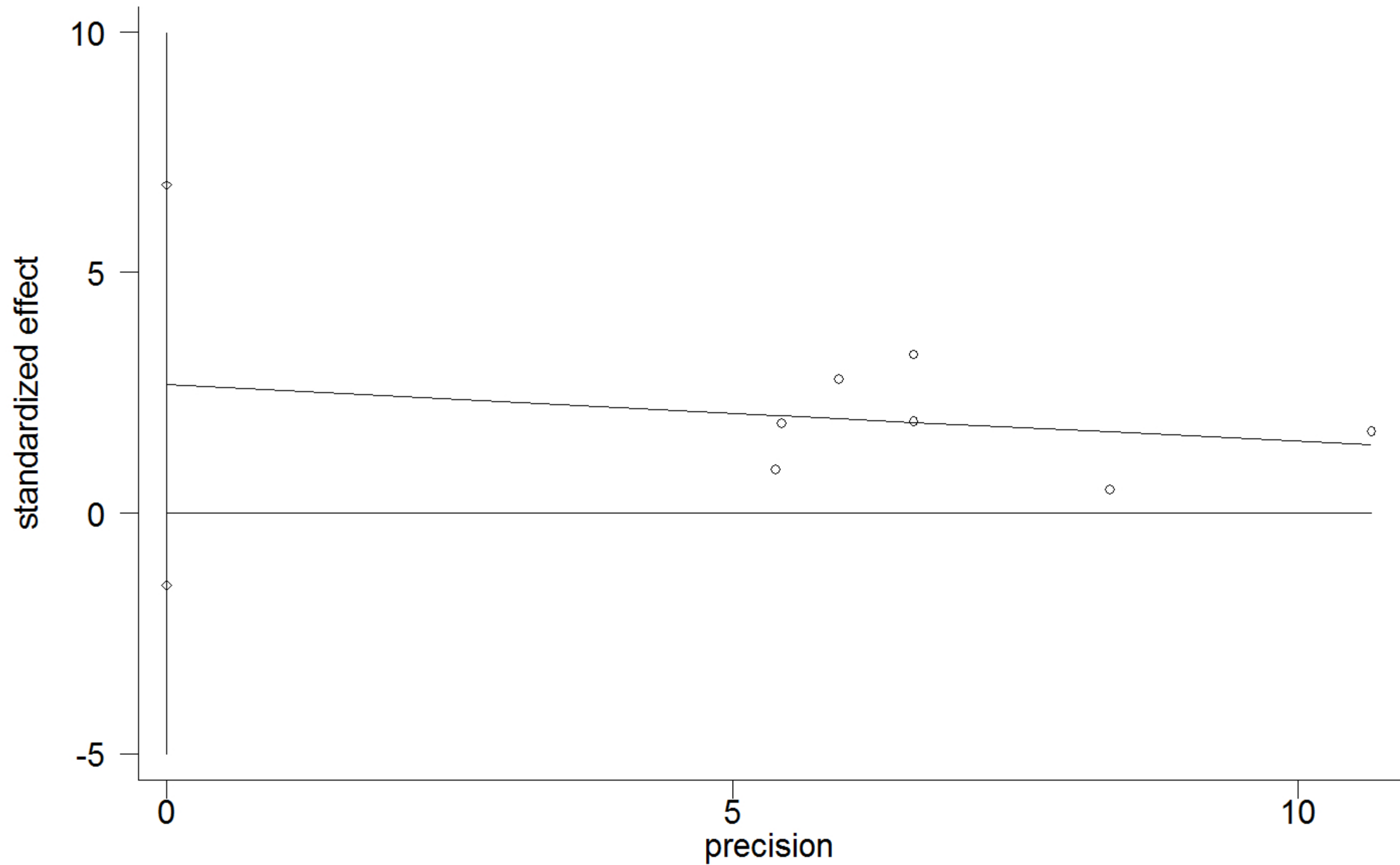

Supplement: Supplementary 6 — Supplementary Figure S6: Egger's publication bias plot of the improvement of the quality of life (PDF). [file 6305645.f6.pdf]

Egger's publication bias plot

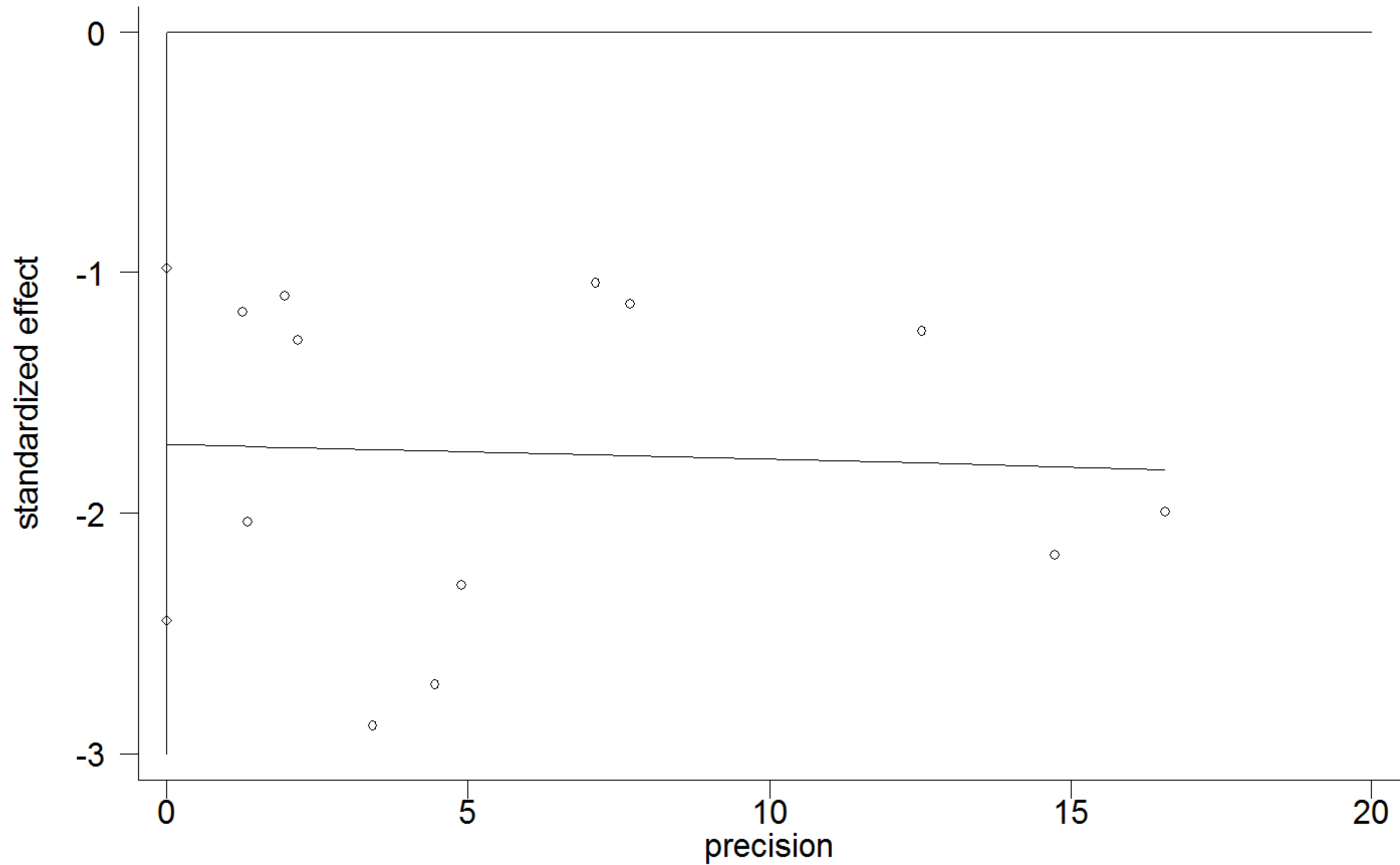

Supplement: Supplementary 7 — Supplementary Figure S7: Egger's publication bias plot of the incidence of decreased WBC count (PDF). [file 6305645.f7.pdf]

Egger's publication bias plot

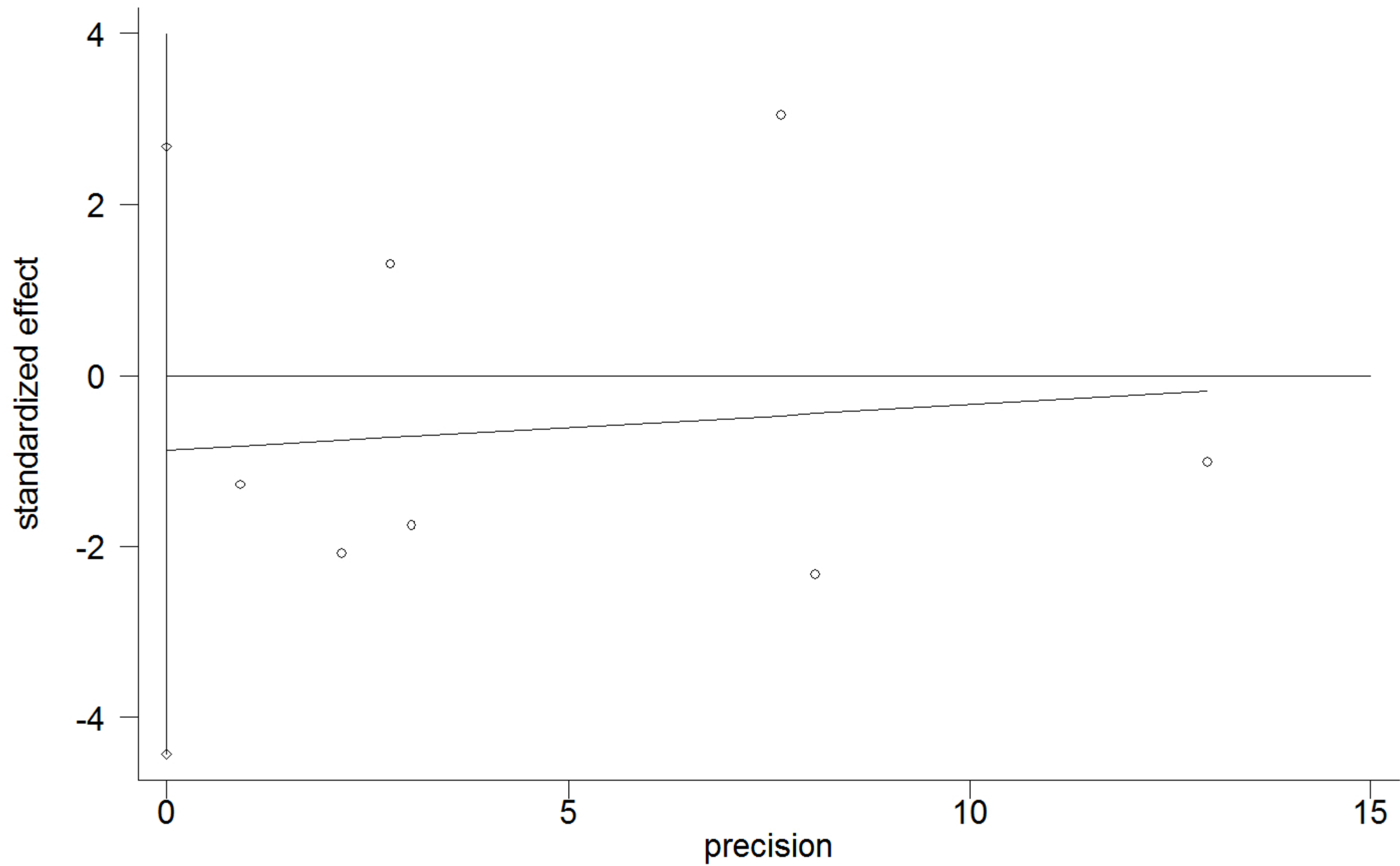

Supplement: Supplementary 8 — Supplementary Figure S8: Egger's publication bias plot of time of the incidence of gastrointestinal adverse reactions (PDF). [file 6305645.f8.pdf]
