# Supplementary material for: The Traditional Chinese Medicine Kangai Injection as an Adjuvant Method in Combination with Chemotherapy for the Treatment of Breast Cancer in Chinese Patients: A Meta-Analysis
Source: Evid Based Complement Alternat Med. 2018 Apr 18;2018:6305645. doi: 10.1155/2018/6305645 (PMC5932437; doi:10.1155/2018/6305645)
Supplement: Supplementary 9 — Supplementary Table 1: search terms (PDF). [file 6305645.f9.pdf]

Supplementary Table 1. Search Terms

Data base	Retrieval strategy
**1. CNKI (Search in All Fields)**	(Thematic = Ruxianai or Thematic = Ruxianzhongliu or Thematic = Xiongbuzhongliu or Thematic = Ruai or Thematic = Ruxianzhongwu or Thematic = Ranfagni or Thematic = Rufangzhongkuai or Thematic = Ruxianzhongkuai or Thematic = Rufangzhongliu) and (Thematic = Kangai) (Exact match)
**2. The VIP information resource integration service platform (VIP) (Search in All Fields)**	(Title or keywords = “Ruxianai” or “Ruxianzhongliu” or “Xiongbuzhongliu” or “Ruai” or “Ruxianzhongwu” or “Rufangai” or “Rufangzhongkuai” or “Ruxianzhongkuai” or “Rufangzhongliu”) and (Title or keywords = “Kangai”)
**3. Wanfang Data knowledge service platform (WanFang Data)**	Title or keywords: (“Ruxianai” + “Ruxianzhongliu” + “Xiongbuzhongliu” + “Ruai” + “Ruxianzhongwu” + “Rufangai” + “Rufangzhongkuai” + “Ruxianzhongkuai” + “Rufangzhongliu”) * Title or keywords: (“Kangai”) * Date: -2017
**4. China Biology Medicine disc (SinoMed)**	#5 (#4) AND (#3)<br>#4 (#2) OR (#1)<br>#3 “Kangai” [Title/Abstract]<br>#2 “Ruxianai” OR “Ruxianzhongliu” OR “Xiongbuzhongliu” OR “Ruai” OR “Ruxianzhongwu” OR “Rufangai" OR “Rufangzhongkuai” OR “Ruxianzhongkuai” OR “Rufangzhongliu” [Title/Abstract]<br>#1 "Ruxianzhongliu"[Mesh]
**5. Pubmed**	#1 Search "Breast Neoplasms"[Mesh]<br>#2 Search (((((((((Breast Neoplasms[Title/Abstract]) OR (Tumors, Breast[Title/Abstract] OR Breast Tumors[Title/Abstract] OR Breast Tumor[Title/Abstract] OR Tumor, Breast[Title/Abstract]

		OR Neoplasms, Breast[Title/Abstract])) OR (Breast Neoplasm[Title/Abstract] OR Neoplasm, Breast[Title/Abstract] OR Breast Cancer[Title/Abstract] OR Cancer, Breast[Title/Abstract] OR Cancer of the Breast[Title/Abstract])) OR (Mammary Cancer[Title/Abstract] OR Cancer, Mammary[Title/Abstract] OR Cancers, Mammary[Title/Abstract] OR Mammary Cancers[Title/Abstract])) OR (Malignant Neoplasm of Breast[Title/Abstract] OR Breast Malignant Neoplasm[Title/Abstract] OR Breast Malignant Neoplasms[Title/Abstract])) OR (Malignant Tumor of Breast[Title/Abstract] OR Breast Malignant Tumor[Title/Abstract] OR Breast Malignant Tumors[Title/Abstract] OR Cancer of Breast[Title/Abstract])) OR (Breast Carcinoma[Title/Abstract] OR Breast Carcinomas[Title/Abstract] OR Carcinoma, Breast[Title/Abstract] OR Carcinomas, Breast[Title/Abstract])) OR (Mammary Carcinoma, Human[Title/Abstract] OR Carcinoma, Human Mammary[Title/Abstract] OR Carcinomas, Human Mammary[Title/Abstract])) OR (Human Mammary Carcinomas[Title/Abstract] OR Mammary Carcinomas, Human[Title/Abstract] OR Human Mammary Carcinoma[Title/Abstract])) OR (Mammary Neoplasms, Human[Title/Abstract] OR Human Mammary Neoplasm[Title/Abstract] OR Human Mammary Neoplasms[Title/Abstract])) OR (Neoplasm, Human Mammary[Title/Abstract] OR Neoplasms, Human Mammary[Title/Abstract] OR Mammary Neoplasm, Human[Title/Abstract])
	#3	Search (Kangai[Title/Abstract] OR Kang'ai[Title/Abstract])

	#4	Search ("Breast Neoplasms"[Mesh]) OR (((((((((((Breast Neoplasms[Title/Abstract]) OR (Tumors, Breast[Title/Abstract] OR Breast Tumors[Title/Abstract] OR Breast Tumor[Title/Abstract] OR Tumor, Breast[Title/Abstract] OR Neoplasms, Breast[Title/Abstract])) OR (Breast Neoplasm[Title/Abstract] OR Neoplasm, Breast[Title/Abstract] OR Breast Cancer[Title/Abstract] OR Cancer, Breast[Title/Abstract] OR Cancer of the Breast[Title/Abstract])) OR (Mammary Cancer[Title/Abstract] OR Cancer, Mammary[Title/Abstract] OR Cancers, Mammary[Title/Abstract] OR Mammary Cancers[Title/Abstract])) OR (Malignant Neoplasm of Breast[Title/Abstract] OR Breast Malignant Neoplasm[Title/Abstract] OR Breast Malignant Neoplasms[Title/Abstract])) OR (Malignant Tumor of Breast[Title/Abstract] OR Breast Malignant Tumor[Title/Abstract] OR Breast Malignant Tumors[Title/Abstract] OR Cancer of Breast[Title/Abstract])) OR (Breast Carcinoma[Title/Abstract] OR Breast Carcinomas[Title/Abstract] OR Carcinoma, Breast[Title/Abstract] OR Carcinomas, Breast[Title/Abstract])) OR (Mammary Carcinoma, Human[Title/Abstract] OR Carcinoma, Human Mammary[Title/Abstract] OR Carcinomas, Human Mammary[Title/Abstract])) OR (Human Mammary Carcinomas[Title/Abstract] OR Mammary Carcinomas, Human[Title/Abstract] OR Human Mammary Carcinoma[Title/Abstract])) OR (Mammary Neoplasms, Human[Title/Abstract] OR Human Mammary Neoplasm[Title/Abstract] OR Human Mammary Neoplasms[Title/Abstract])) OR (Neoplasm, Human

	Mammary[Title/Abstract] OR Neoplasms, Human Mammary[Title/Abstract] OR Mammary Neoplasm, Human[Title/Abstract]))<br>#5 #3 OR #4
**6. Cochrane library**	#1 MeSH descriptor: [Breast Neoplasms] explode all trees<br>#2 Breast Neoplasms:ti,ab,kw OR Tumors, Breast:ti,ab,kw OR Breast Tumors:ti,ab,kw OR Breast Tumor:ti,ab,kw OR Tumor, Breast:ti,ab,kw OR Neoplasms, Breast:ti,ab,kw OR Breast Neoplasm:ti,ab,kw OR Neoplasm, Breast:ti,ab,kw OR Breast Cancer:ti,ab,kw OR Cancer, Breast:ti,ab,kw OR Cancer of the Breast:ti,ab,kw OR Mammary Cancer:ti,ab,kw OR Cancer, Mammary:ti,ab,kw OR Cancers, Mammary:ti,ab,kw OR Mammary Cancers:ti,ab,kw OR Malignant Neoplasm of Breast:ti,ab,kw OR Breast Malignant Neoplasm:ti,ab,kw OR Breast Malignant Neoplasms:ti,ab,kw OR Malignant Tumor of Breast:ti,ab,kw OR Breast Malignant Tumor:ti,ab,kw OR Breast Malignant Tumors:ti,ab,kw OR Cancer of Breast:ti,ab,kw OR Breast Carcinoma:ti,ab,kw OR Breast Carcinomas:ti,ab,kw OR Carcinoma, Breast:ti,ab,kw OR Carcinomas, Breast:ti,ab,kw OR Mammary Carcinoma, Human:ti,ab,kw OR Carcinoma, Human Mammary:ti,ab,kw OR Carcinomas, Human Mammary:ti,ab,kw OR Human Mammary Carcinomas:ti,ab,kw OR Mammary Carcinomas, Human:ti,ab,kw OR Human Mammary Carcinoma:ti,ab,kw OR Mammary Neoplasms, Human:ti,ab,kw OR Human Mammary Neoplasm:ti,ab,kw OR Human Mammary Neoplasms:ti,ab,kw OR Neoplasm, Human Mammary:ti,ab,kw OR Neoplasms, Human Mammary:ti,ab,kw OR Mammary Neoplasm, Human:ti,ab,kw<br>#3 kangai:ti,ab,kw or kang'ai:ti,ab,kw (Word variations have been

	searched)<br>#4 #1 or #2<br>#5 #3 and #4
**7. Embase**	#1 ' kangai':ab,ti OR ' kang'ai':ab,ti<br>#2 Search 'Breast Neoplasms’:ab,ti OR 'Tumors, Breast’:ab,ti OR 'Breast Tumors’:ab,ti OR 'Breast Tumor’:ab,ti OR 'Tumor, Breast’:ab,ti OR 'Neoplasms, Breast’:ab,ti OR 'Breast Neoplasm’:ab,ti OR 'Neoplasm, Breast’:ab,ti OR 'Breast Cancer’:ab,ti OR 'Cancer, Breast’:ab,ti OR 'Cancer of the Breast’:ab,ti OR 'Mammary Cancer’:ab,ti OR 'Cancer, Mammary’:ab,ti OR 'Cancers, Mammary’:ab,ti OR 'Mammary Cancers’:ab,ti OR 'Malignant Neoplasm of Breast’:ab,ti OR 'Breast Malignant Neoplasm’:ab,ti OR 'Breast Malignant Neoplasms’:ab,ti OR 'Malignant Tumor of Breast’:ab,ti OR 'Breast Malignant Tumor’:ab,ti OR 'Breast Malignant Tumors’:ab,ti OR 'Cancer of Breast’:ab,ti OR 'Breast Carcinoma’:ab,ti OR 'Breast Carcinomas’:ab,ti OR 'Carcinoma, Breast’:ab,ti OR 'Carcinomas, Breast’:ab,ti OR 'Mammary Carcinoma, Human’:ab,ti OR 'Carcinoma, Human Mammary’:ab,ti OR 'Carcinomas, Human Mammary’:ab,ti OR 'Human Mammary Carcinomas’:ab,ti OR 'Mammary Carcinomas, Human’:ab,ti OR 'Human Mammary Carcinoma’:ab,ti OR 'Mammary Neoplasms, Human’:ab,ti OR 'Human Mammary Neoplasm’:ab,ti OR 'Human Mammary Neoplasms’:ab,ti OR 'Neoplasm, Human Mammary’:ab,ti OR 'Neoplasms, Human Mammary’:ab,ti OR 'Mammary Neoplasm, Human’:ab,ti<br>#3 #1 and #2
