# Supplementary material for: The Traditional Chinese Medicine Kangai Injection as an Adjuvant Method in Combination with Chemotherapy for the Treatment of Breast Cancer in Chinese Patients: A Meta-Analysis
Source: Evid Based Complement Alternat Med. 2018 Apr 18;2018:6305645. doi: 10.1155/2018/6305645 (PMC5932437; doi:10.1155/2018/6305645)

**Supplementary Table 4: The publication bias results of disappearance of** **incidence of WBC count decrease with Egger’s test**

**(A) The result of incidence of WBC count decrease with Egger's test**


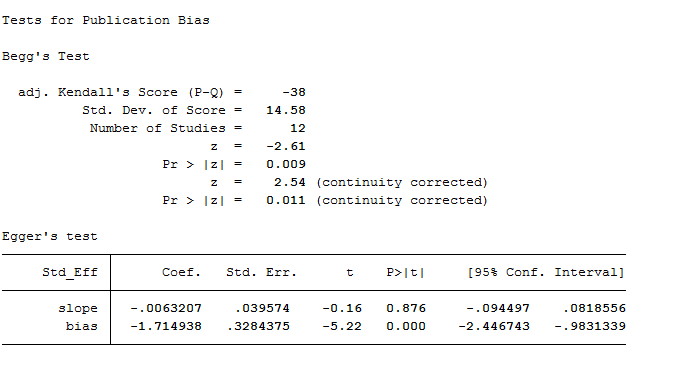


**(B) Trim and filling method with soft Stata 12.0**


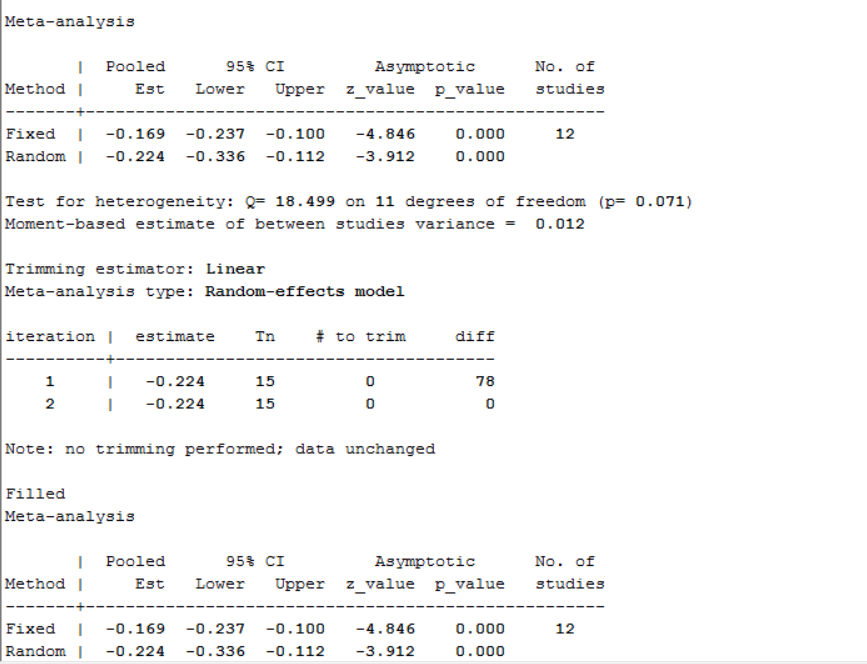

Supplement: Supplementary 12 — Supplementary Table 4: the publication bias results of disappearance of incidence of WBC count decrease with Egger's test (DOCX). [file 6305645.f12.docx]
